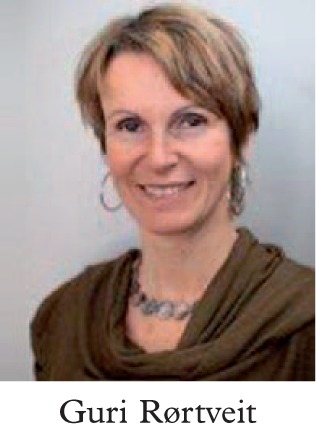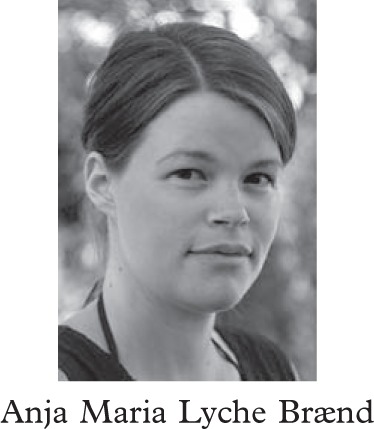# A full Norwegian editorial change. Two men replaced by two women

**DOI:** 10.3109/02813432.2014.900312

**Published:** 2014-03

From 2014, the Scandinavian Journal of Primary Health Care's Editorial Board will have the honour of welcoming a new national and a new assistant editor from Norway. And at the same time we must say goodbye to the two former Norwegian editors.

## The former Norwegian editors

In 1999, Scandinavian Journal of Primary Health Care welcomed Anders Bærheim as national editor and Morten Lindbæk as assistant editor. Anders is a GP and Professor at Department of Global Public Health and Primary Care, University of Bergen, Norway. Anders has been a very important person in the editorial board. Anders has always ensured the scientific level and always done this in his kind, firm and tactful way. In an editorial board, this talent is one of the most important to have. Many authors will also acknowledge Anders for his ‘midwifery’ role. He could see a potential in a paper, and then he assisted the authors on the way to publication. Anders also has been very important in defining the editorial strategy and has been the driving force for the qualitative research publication in the Scandinavian Journal of Primary Health Care. We thank Anders for all his efforts and friendship.

Morten Lindbæk is a GP and Professor at Department of General Practice, Institute of Health and Society, University of Oslo. Morten has since 1999 managed a huge number of manuscripts. He has an impressive track-record and really knows about publishing medical research. Morten's fantastic network and knowledge about clinical family medicine has been a perfect input and part of the editorial board. We thank Morten for all the reviews, assessments and inputs.

## The new Norwegian editors

From 2014, after 15 years with Anders and Morten, two women are taking over. Guri Rørtveit is a GP and Professor, research leader at Department of Global Public Health and Primary Care, University of Bergen. Guri enters the Editorial board as the new Norwegian national editor. Being a research director with a large professional network, Guri will be central in supporting and enhancing the positive development of the Scandinavian Journal of Primary Health Care. Professor Rørtveit's research area includes infectious disease, epidemiologic studies and women's health. These are all very central aspects of academic family medicine. We know that the Norwegian researchers are looking very much forward to working with Guri.

As new assistant editor in Norway, we have the pleasure of welcoming Anja Maria Lyche Brænd. She is MD and works as a PhD research fellow at Department of General Practice, Institute of Health and Society, University of Oslo. Anja Maria works with medical education and general practice.

On behalf of the Scandinavian Journal of Primary Health Care's Editorial Board, I wish to express my honest and sincerest thanks to Anders and Morten, and to give the warmest welcome to Guri and Anja Maria.

Tusen takk for flott innsats!!

Peter Vedsted

Editor-in-Chief